# Pollen-borne microbes shape bee fitness

**DOI:** 10.1098/rspb.2018.2894

**Published:** 2019-06-12

**Authors:** Prarthana S. Dharampal, Caitlin Carlson, Cameron R. Currie, Shawn A. Steffan

**Affiliations:** 1 Department of Entomology, University of Wisconsin, Madison, WI, USA; 2 Department of Bacteriology, University of Wisconsin, Madison, WI, USA; 3 USDA-ARS, Vegetable Crops Research Unit, Madison, WI, USA

**Keywords:** bee larval survivorship, microbes, pollen provisions, trophic position, trophic biomarker analysis

## Abstract

Teeming within pollen provisions are diverse communities of symbiotic microbes, which provide a variety of benefits to bees. Microbes themselves may represent a major dietary resource for developing bee larvae. Despite their apparent importance in sustaining bee health, evidence linking pollen-borne microbes to larval health is currently lacking. We examined the effects of microbe-deficient diets on the fitness of larval mason bees. In a series of diet manipulations, microbe-rich maternally collected pollen provisions were replaced with increasing fractions of sterilized, microbe-deficient pollen provisions before being fed to developing larvae. Convergent findings from amino acid and fatty acid trophic biomarker analyses revealed that larvae derived a substantial amount of nutrition from microbial prey and occupied a significantly higher trophic position than that of strict herbivores. Larvae feeding on increasingly sterile diets experienced significant adverse effects on growth rates, biomass and survivorship. When completely deprived of pollen-borne microbes, larvae consistently exhibited marked decline in fitness. We conclude that microbes associated with aged pollen provisions are central to bee health, not only as nutritional mutualists, but also as a major dietary component. In an era of global bee decline, the conservation of such bee–microbe interactions may represent an important facet of pollinator protection strategies.

## 1. Background

Bees are the predominant group of insect pollinators on the Earth. Widespread declines in managed and wild bee populations have major implications for global food security and ecosystem stability [1,2]. Among several other well-known factors (e.g. habitat loss, decreased genetic diversity), the dramatic increase in the use of pesticides (e.g. neonicotinoids) has been directly linked to unprecedented bee losses [3–5]. Such findings have restricted the use of many hazardous insecticides, although other agrochemicals (fungicides and herbicides) that pose low risk of direct toxicity for adult bees, continue to be sprayed on in-bloom crops without much scrutiny [6,7]. While relatively non-toxic for the adults (as measured by LD_50_ in honeybees), it is hard to explain why such ‘bee-safe’ compounds cause persistent and widespread decline among larval bees [8].

Nearly all metazoans [9], including bees, are associated with a diverse community of microbes, commonly known as the microbiome. The microbiome of both social [10] and solitary bees [11–14] consists of various microbial symbionts, wherein the microbes may engage in mutualistic, commensalistic and/or parasitic interactions with their insect host [15]. Collectively referred to as the bee–microbe symbioses, these ecological interactions have a crucial impact on bee health [16]. Nutritional mutualism with their gut microbiome is especially critical for adult social bees [17–19]. Larval bees, however, lack the characteristic gut microbiome needed to digest raw pollen and instead rely on consortia of external pollen-borne microbial symbionts to meet their nutritional requirements [20]. Past research using bumblebees suggest that alterations to the microbial community within hive-stored pollen can potentially disrupt this critical symbiosis [21], possibly explaining the high mortality among larvae when exposed to agrochemicals that are deemed safe for adult bees [22].

Pollen provisions of social [21,23–25] and solitary bees [12–14,16,26,27] are replete with specialized non-pathogenic microbes, which ferment and consume the raw pollen, improving its digestibility, ‘shelf-life’ and nutritional content [23–25,28–31]. This microbe-mediated fermentation transforms the recalcitrant pollen substrate into a nutrient-dense mixture of pre-digested pollen, honey, nectar and diverse microbes that subsequently serves as the primary diet for developing larvae [13,14,16,21,24,29,31–36]. Specific microbial taxa within pollen provisions are also known to aid in the long-term preservation of stored pollen, produce vital macromolecules and provide protection against parasites and pathogens [37,38]. Interestingly, past work in honeybees shows that the microbial community of hive-stored pollen is distinct from that of the bee gut [39]. This suggests that for honeybees, these two microbial communities perform unique functions within their respective microhabitats and are not interchangeable. Altogether, the community of pollen-borne microbes within an ageing, fermenting pollen mass provides larval bees with a broad range of developmental benefits, and is therefore, related to bee fitness [40–43].

Solitary bees represent nearly 85% of all bee species and are significant pollinators for wild and managed crops [44]. Unlike social bees, solitary bees do not form colonies; instead, each female mates independently and builds her own nest. Within the nest are individually partitioned brood chambers, each stocked with a finite mass of maternally collected pollen and nectar, and a single egg. Also enmeshed within the pollen provision, is a diverse and biologically important microbial community obtained from various sources including pollen, nectar, adult forager and/or nest building material [13]. Inside each brood chamber, microbes colonize, digest and consume the raw pollen and nectar components [26,27,36,45,46]. As the pollen–nectar provision ages, these microbes become thoroughly integrated within the plant-based substrate. Therefore, when a developing bee larva consumes the aged provision, it probably ingests the plant-based components (pollen and nectar) and the plant-eating microbes (herbivorous organisms). This implies that larval bees acquire food from multiple trophic levels, and therefore, are omnivorous. Given the ubiquity of microbes within pollen provisions [21,23,35,47], such omnivory is probably unavoidable, perhaps even beneficial for larval bees. Indeed, recent findings suggest that nearly all bees consume substantial amounts of non-plant proteins, probably derived from microbial prey [48]. As nutritional mutualists and as prey items, these microbes represent both symbionts and prey for bees—the pollen-borne microbes appear to be cultured by bees much in the same way that leaf-cutter ants cultivate symbiotic fungi [49,50]. These microbial communities may strongly influence larval development in solitary bees that do not receive extensive brood care (e.g. trophallaxis, social tending and incremental feeding), and rely entirely on the microbes within the maternal provisions [51]. However, to date, there is no quantitative evidence linking microbes within pollen provisions and larval fitness in solitary bees.

Because microbial trophic interactions are exceedingly complex, empirical evidence integrating microbes within the trophic hierarchy has been hard to obtain [52]. However, recent findings from compound specific isotopic analysis of amino acids (CSIA), an advanced biomarker-based assay, have unveiled the trophic function of microbes [53]. Estimations of microbial trophic position (TP) using the *δ*^15^N signature of canonical ‘source’ (i.e. phenylalanine; *δ*^15^N_phe_) and ‘trophic’ (i.e. glutamic acid; *δ*^15^N_glu_) amino acids reveal that heterotrophic microbes serve as prey for countless higher-order consumers [54]. Fatty acids represent another class of biomarkers that have been commonly used to describe microbial trophic ecology. This technique is based on the principle that consumers directly incorporate fatty acids from their diet into their own storage lipids (neutral lipids) without modification [55]. Because neutral lipid fatty acids (NLFA) within consumer biomass closely reflect the fatty acid profile of their diet, prey-specific fatty acid biomarkers have been widely applied in dietary reconstruction studies [56–60]. While there is growing evidence indicating the dominance of microbial prey for numerous metazoan consumers [61–63], it has only recently been documented in bees [48].

In order to better conserve solitary bee populations, a more refined understanding of the trophic function of their pollen-borne symbionts is needed. To this end, we use a combined approach of diet manipulations and advanced trophic biomarker-based assays to investigate the effects of excluding pollen-borne microbes on the health of solitary bee larvae. We hypothesize that excluding these microbes will have measurable adverse effects on larval health, demonstrated by slower growth rates, lower biomass, and lower survivorship. We use an *in vitro* diet manipulation experiment wherein solitary bee larvae are reared on a gradient of microbe-deficient pollen provisions. Next, we merge two independent trophic biomarker-based techniques to examine the link between dietary patterns and larval health. First, we use CSIA to estimate larval TP based on the *δ*^15^N signature of glutamic acid and phenylalanine [54]. As an autotrophic substrate, the TP of pollen is approximately 1.0 [48], and if bee larvae solely consume a pollen-based diet, then as strict herbivores, their TP should register at approximately 2.0 (i.e. the expected TP of strict herbivores). Any significant elevation above 2.0 would indicate that the larvae assimilated proteins from heterotrophic (non-plant) prey. Second, we characterize larval NLFA profiles to identify larval feeding strategies using known biomarkers for bacterial, fungal and autotrophic resources [56]. Through these multiple lines of inquiry, we converge on the contribution and impact of microbes as a dietary subsidy for larval bees.

## 2. Methods

### (a) Bees

In April 2017, freshly plugged *Osmia ribifloris* nesting reeds were obtained in a single overnight shipment from a commercial supplier (NativeBees.com). All reeds were collected from a single location in Kaysville, Utah, where the adult females foraged on nectar and pollen from the surrounding unmanaged vegetation. Reeds were dissected in the laboratory using a sterilized razor, and the eggs were sexed based on cell size, position and volume of provision. *Osmia* sp. allocate smaller pollen provisions to the male offspring, resulting in lower body weights compared with that of females [64]. To avoid such gender-bias, only the male eggs were used in this study. The eggs were separated from the pollen provisions with a clean paintbrush and randomized across treatments.

### (b) Pollen provisions

The fresh weight of pollen provisions from each male cell was recorded and then pooled into a single mass to reduce any bias from maternal provisioning and genetic relatedness. Half of this collected mass was freeze-dried, soaked in 95% ethanol and dried under germicidal ultraviolet light in a biosafety cabinet overnight. The remaining untreated half represented natural, i.e. non-sterile pollen (NSP). Quantitative analysis of macronutrients and minerals (outsourced to University of Wisconsin, Marshfield) was used to confirm that the sterilization process did not compromise the nutritional composition of the pollen provision (electronic supplementary material, table S1). Previous studies indicate that this method of sterilization has no effect on the TP of the substrate [53,61], and does not have a significant impact on the nutritional quality of pollen [21].

### (c) Experimental design

The experiment consisted of seven treatments, varying based on the fraction of sterile (SP) and natural, i.e. NSP (100%, 90%, 80%, 70%, 60%, 50% and 0%SP). Each treatment consisted of 12 replicates (*n* = 12) and was conducted in a separate 48-well plate using previously published methods [65] (electronic supplementary material, figure S1). Sterile fractions were rehydrated with appropriate volumes of sterile water to replicate the moisture content of natural provisions. Pollen provisions were reconstituted by aseptically mixing appropriate weights of rehydrated SP with NSP, such that the end weight of the reconstituted provision was the same as originally allocated to the male provisions. A randomly selected egg was then placed into a well lined with a sterilized tin cup containing the pollen provision. The plates were loosely taped and maintained under dark conditions at 22°C. Larvae were allowed to develop until they reached the prepupal stage, characterized by the completion of a pale silken cocoon.

### (d) Data collection

The larvae were observed daily to record survivorship. To minimize handling stress and reduce the risk of environmental contamination, weights of all surviving larvae were recorded aseptically on days 1, 10, 15 and 20 within a biosafety cabinet. The following larval response variables were recorded for all treatments: survivorship, larval and prepupal fresh weight, and larval developmental time. Larval survivorship was explored using Kaplan–Meier survival plots and log-rank tests, pooled over strata. A repeated measure ANOVA was used to test the differences between larval weights across seven treatments and four time points and was followed by pairwise comparisons. Prepupal biomass was compared using one-way ANOVA followed by Tukey's post hoc test. Larval developmental time was compared using Kruskal–Wallis non-parametric ANOVA. Pearson correlation analysis was used to explore the relationship between (i) prepupal biomass and (ii) larval developmental time and %SP of pollen provisions.

### (e) Trophic biomarker analysis

CSIA of *δ*^15^N amino acids of randomly selected larvae raised on 100%SP and 0%SP (*n* = 3 each) was outsourced to the Japan Agency for Marine-Earth Science and Technology. Briefly, larvae were hydrolysed using HCl and derivatized with thionyl chloride/isopropanol and pivaloyl chloride/dichloromethane. The derivatives were then analysed using a gas chromatography-combustion-isotope ratio mass spectrometer to quantify their isotopic signatures. We estimated larval TP using previously established equations for terrestrial, C3 plant-based food-webs [53,66]; TP = [(*δ*^15^N_glu_ – *δ*^15^N_phe_ + 8.4)/7.2] + 1. Larval TP estimates of both groups were compared to a test value of TP_herbivore_ = 2.0 using one-sample one-tailed *t*-tests*.* TP estimates of larvae raised on 100%SP and 0%SP were compared to each other using independent samples *t-*test.

NLFA analysis of randomly selected larvae raised on 100%SP and 0%SP (*n* = 5 each) was outsourced to the University of Alabama. Briefly, larval lipids were extracted using a 1 : 2 : 0.6 (v/v/v) mixture of dichloromethane-methanol-50 mM phosphate buffer (pH 7.4) solution. After 24 h at 4°C, the solution was split using 1 : 1 (v/v) dichloromethane and deionized water to collect the organic phase containing total lipids. Neutral lipids were separated from total lipids by silica gel solid phase extraction and converted to their fatty acid methyl esters (FAMEs) by base methanolysis and purified by octadecyl bonded silica gel (C18) reverse-phase column chromatography. FAMEs were analysed using gas chromatographic flame ionization detection and identified by co-elution with known standards and mass spectral analysis [60,67]. Total and relative abundance of NLFAs were estimated from the chromatograms. NLFA profiles were compared using principal component analysis (PCA) after transforming the data to the natural log (ln (*x* + 1)) and visualized using a scatter plot to assess separation between the two treatment groups. Bacterial contribution to larval diet was calculated using the sum of previously identified absolute bacterial biomarkers (a15:0, i15:0, a17:0, i17:0). The contributions of plants and fungi were calculated based on the abundance of relative plant- and fungi-specific biomarkers (16:1*ω*13t, 18:1*ω*9, 18:2*ω*6). The ratio of relative biomarkers 18:2*ω*6 and 18:1*ω*9 (18:2*ω*6/18:1*ω*9) of both groups was compared using an independent sample *t*-test to distinguish between fungivorous and herbivorous feeding strategies [56,59,68]. Total abundance of de novo synthesized free fatty acids was estimated from the sum of 12:0, 14:1, 14:0, 16:1, 16:0 and 18:1 and compared across the two groups using a Mann–Whitney *U*-test. All analyses were conducted using SPSS 23.0, IBM, Chicago, Illinois.

## 3. Results

**Figure 1.**
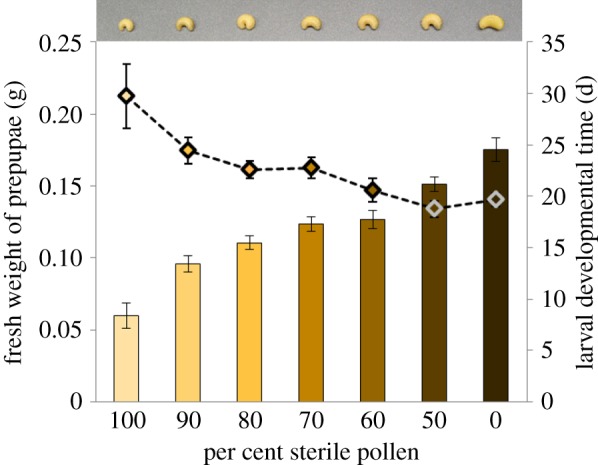
Per cent sterile pollen within larval diet had a significant impact on prepupal biomass (*p* < 0.001) (solid bars), and larval developmental time (*p* < 0.001; dotted line; mean ± 1 s.e.). Inset panel above shows the difference in biomass among representative prepupae corresponding to each of the seven treatments. *note:* All bees are at the same developmental stage, i.e. the prepupal stage.

**Figure 2.**
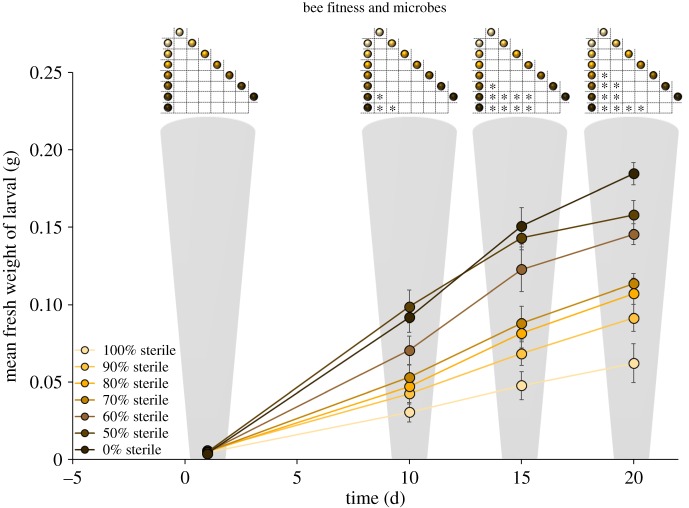
Larval fresh weights measured over four time points across seven diet treatments. Greenhouse–Geisser corrected results from repeated measures show significant effects of time, (*p* < 0.0001), treatment (*p* < 0.0001) and time × treatment (*p* < 0.0001) on larval fresh weight. Inset figures represent pairwise comparisons of larval weights across diet treatments within a given time increment (*p < 0.05).

Survival analysis using the Kaplan–Meier log-rank test followed by pairwise comparisons indicated that survivorship was significantly lower among treatments with higher %SP (Bonferroni-corrected *p* < 0.001; electronic supplementary material, figures S4, S5 and table S3). A repeated measure ANOVA was used to analyse larval weight gain across seven treatments and four time points. Greenhouse–Geisser correction (*ɛ* = 0.59) was used to adjust for the violation of Mauchly's test of sphericity ($\chi_{5}^{2}=47.74$ , *p* < 0.001). Results showed a significant main effect of time, (*F*_1.77, 72.62_ = 494.92, *p* < 0.0001), and treatment (*F*_6, 41_ = 13.14, *p* < 0.0001). There was also a significant interaction between time and treatment (*F*_10.63, 72.62_ = 11.26, *p* < 0.0001). Pairwise comparisons following this interaction indicated that while there was no difference in larval weight across treatments on day 1, larvae consuming higher %SP showed lower weight gain as time progressed (figure 2). The %SP in diet had a significant effect on the fresh weight of prepupae (one-way ANOVA, *F*_6, 59_ = 32.97, *p* < 0.0001) and Tukey post hoc tests indicated that larvae raised on 100%SP had significantly lower prepupal biomass than all other treatments. Higher %SP in pollen provisions significantly prolonged larval developmental time (Kruskal–Wallis, *H*_6_ = 26.53, *p* < 0.0001; figure 1; electronic supplementary material, table S3). Appropriate pairwise comparisons of survivorship, prepupal biomass and developmental time indicated significant differences across several treatment pairs, which followed a predictable pattern; i.e. survivorship and biomass was predictably lower, and developmental time was predictably higher among treatments consuming higher fractions of SP (electronic supplementary material, figure S4). Pearson correlation analysis showed that the %SP within pollen provisions had a significant negative relationship with prepupal fresh weight (*r* = −0.82, *n* = 66, *p* < 0.0001), and a significant positive relationship with larval developmental time (*r* = 0.48, *n* = 66, *p* < 0.0001; electronic supplementary material, figure S3).

**Figure 3.**
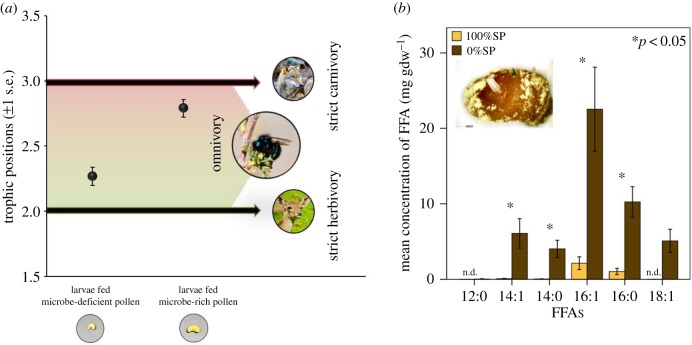
(*a*) Trophic positions (mean ± s.e.) of larvae raised on microbe-deficient (100%SP) and microbe-rich (0%SP) pollen (*n* = 3 each). Trophic positions of larvae from both treatments were significantly higher than that of strict herbivores (=2.0). Trophic position of larvae raised on microbe-rich pollen was significantly higher than that of larvae raised on microbe-deficient pollen (*p* = 0.006). Inset panel below shows representative prepupae corresponding to the two treatments at the same scale. High-resolution image of an adult *O. ribifloris* courtesy of Dr Jim Cane. Photo source: strict herbivore, Ural meridian; strict carnivore, Arne von Brill/Flickr. (*b*) Concentration of free fatty acids (FFAs) from larvae raised on microbe-deficient (100%SP) and microbe-rich (0%SP) pollen (mean ± 1 s.e.; *n* = 5 per treatment) (n.d. = not detected). Inset panel represents a single natural pollen provision and the associated egg of *O. ribifloris* collected from nesting reeds.

TP of larvae consuming 100%SP and 0%SP was 2.27 ± 0.07 and 2.79 ± 0.07, respectively (mean ± 1 s.e.). TP of larvae from both treatments were significantly higher than expected of strict herbivores (TP_herbivore_ = 2.0; 100%SP: *p* = 0.03; 0%SP: *p* = 0.003). TP of larvae consuming 0%SP was significantly higher compared with larvae consuming 100%SP (figure 3*a*; *p* = 0.006; electronic supplementary material, table S4). Results from the PCA showed that the first principal component (PC1) accounted for 62% of the variability in larval NLFA profiles. Highest positive factor loadings along PC1 were attributed to greater abundance of 18:1*ω*7c, 18:1*ω*9, 14:1b, 18:1*ω*5 and 16:1*ω*13t, whereas highest negative factor loadings were attributed to greater abundance of 18:0, 10:0 and 12:0. A scatter plot obtained from the factor loadings was used to visualize the separation between the larvae fed 100%SP and 0%SP based on NLFA profiles, with 9 out of 10 larvae clustering based on treatment type. All larvae fed 100%SP had positive PC1 factor loadings and clustered together, and 4 out of the 5 larvae fed 0%SP had negative PC1 factor loadings and clustered together. The ratio of relative fungal and plant biomarkers (18:2*ω*6/18:1*ω*9) was significantly lower in larvae fed 100%SP than those fed 0%SP (0.20 ± 0.02 and 0.31 ± 0.02 mg gdw^−1^ respectively) (*p* = 0 .008). The sum of bacterial biomarkers (a15:0, i15:0, a17:0, i17:0) was trivial in both groups (less than 1% of total NLFAs by weight), although the concentration was half in the larvae raised on 100%SP compared to those raised on 0%SP (0.29 ± 0.08 and 0.60 ± 0.46, respectively). Total abundance of free fatty acids (12:0; 14:1; 14:0; 16:1; 16:0; 18:1) was significantly higher among larvae fed 0%SP (240.31 ± 12.13 mg gdw^−1^) compared to larvae fed 100%SP (16.55 ± 1.30 mg gdw^−1^; *p* < 0.01; a detailed list of larval NLFA profile is included in the electronic supplementary material, table S5 and figure S6).

## 4. Discussion

Bees are thought to derive a wide range of benefits from non-pathogenic microbes present within the larval pollen provisions. Removal of these beneficial external symbionts, therefore, would disrupt the suite of microbe-derived services, compromising larval bee development. Our findings offer strong support for this hypothesis, revealing that larval bees are quite dependent on microbes as nutritional, external symbionts. Whether sourced from pollen, nectar and/or the adult forager, the microbial communities in fermenting pollen provisions appear to transform the pollen–nectar blend into a complex of living organisms and plant biomass [48,61,69]. Our data indicate that without the full complement of microbial organisms in the aged provision, solitary bee larvae endure higher mortality and slower growth, suggesting that these larvae are denied adequate nutrition for development. The overall trend across all diet treatments reveal a clear pattern in which the incremental removal of microbes from pollen provisions resulted in significantly smaller larvae, slower growth rates and fewer larvae surviving to pupation (figure 1). These results represent evidence of a nutritional gradient mediated by pollen-borne microbes.

At the start of the study, all bee larvae hatched as healthy neonates, fed on ample pollen provisions and grew—indeed, across all treatments, the larvae showed a significant increase in biomass over time. However, while their initial weights were comparable, by day 10, there were significant differences in the biomass of larvae raised on diets representing the ends of the treatment gradient, i.e. larval biomass was significantly lower among larvae consuming microbe-deficient pollen, compared to those consuming higher fractions of microbe-rich pollen. Pairwise comparisons across all seven treatments and four time points revealed that this disparity in growth rate continued to widen predictably over time until the prepupal stage (figure 2). The fraction of microbe-deficient pollen also had a significantly negative impact on prepupal biomass. Furthermore, as pollen-borne microbes were progressively removed from their diet, larvae took increasingly longer to develop, and ultimately had lower prepupal biomass. Consuming the microbe-deficient pollen diets resulted in a statistically significant decline in larval survivorship across all treatments. It should be emphasized here that the dying bee larvae had ample pollen provisions to eat—as much pollen as the larvae that were thriving on microbe-rich provisions. Of the survivors, larvae fed completely sterile pollen (100%SP) took approximately 1.5 times longer to reach the prepupal stage and weighed approximately 66% less than those raised on natural pollen (0%SP). Pairwise comparisons indicated this pattern of declining fitness strongly paralleled the fraction of microbe-deficient pollen in larval diet, i.e. fitness was lowest for larvae raised on completely sterile pollen, increased predictably with higher fractions of microbe-rich pollen in the intermediate treatments, and was highest for larvae raised on completely natural pollen.

As dominant consumers of nectar and pollen, bees have long been considered strictly herbivorous. However, suffused throughout the aged pollen provisions are diverse microbes that ferment, consume and assimilate plant-based proteins that are otherwise inaccessible to larval bees. This microbial fermentation transforms the raw pollen into a mixture of microbial and plant biomass, and when feeding on this detrital complex, larval consumers ingest varying proportions of microbes along with the entire detrital mass, assimilating proteins of both heterotrophic (microbial) and autotrophic (plant) origin [61]. Indeed, recent evidence suggests that most (if not all) bees assimilate proteins from heterotrophic pollen-borne microbes, often in amounts that exceed contributions from pollen-based protein [48]. This implies that removing microbes from pollen provisions would deprive larvae of a major dietary component.

Findings from amino acid trophic biomarker analysis revealed that larval trophic position was significantly more elevated than that expected of strict herbivores, and that the larvae were in fact, omnivorous. Because microbes are trophic analogues of metazoan consumers, pollen-eating microbes are, functionally, herbivores [52,53]. When consuming the microbe-rich pollen provisions, bee larvae derived dietary proteins from both trophic levels 1 (pollen) and 2 (herbivorous microbes), and therefore registered as omnivores rather than strict pollen-feeding herbivores. However, as expected, the magnitude of microbial dietary subsidy was significantly lower among larvae raised on sterile pollen (approx. 30%), compared to those raised on natural pollen (approx. 80%). Although the diet of the former group was initially devoid of microbes, the sterile pollen was probably recolonized (albeit slowly) over the course of the experiment. Nonetheless, the removal of microbes early in the larval developmental phase dramatically reduced the availability of a vital dietary resource. This would explain the significant reduction in trophic position for larvae consuming sterile pollen, compared to those consuming pollen replete with microbial prey (figure 3*a*).

Dietary reconstruction using NLFA trophic biomarkers revealed that along with pollen, larval bees consumed measurable amounts of microbial prey during development. However, results from the PCA analysis showed distinct differences in the abundance of key NLFA biomarkers within larval biomass based on diet treatments. Larvae raised on sterile pollen had higher relative abundances of plant-associated NLFA biomarkers (16:1*ω*13t, 18:1*ω*9), suggesting greater herbivory within this group [56,59,70,71]. Similar trends were noted from the ratio of relative fungal : plant biomarkers (18:2*ω*6/18:1*ω*9), which was significantly lower among larvae fed sterile pollen compared with those fed natural pollen. These findings suggest that when microbes were absent from their diet, larval bees showed a herbivorous feeding strategy, relying more on a plant-based diet. By contrast, larvae feeding on the microbe-rich natural pollen were more fungivorous [56,59,67,68]. Additionally, the abundance of free fatty acids (12:0; 14:1; 14:0; 16:1; 16:0; 18:1), which serve as the primary source of energy during non-feeding periods [72], was nearly 15 times higher among larvae raised on natural pollen, suggesting greater fitness for this group [73] (figure 3*b*). These results indicate that microbes within pollen provisions formed an essential dietary component for larvae, and when microbes were reduced and/or excluded from their diet, larvae showed greater dependence on pollen-based nutrients. Together with our earlier results, we conclude that larval bees assimilated significant microbe-derived proteins and lipids during development, and the absence of pollen-borne microbes had a detrimental effect on larval fitness.

Despite the strong evidence linking pollen-borne microbes and bee fitness, it may be argued that the trends seen in our study could be owing to alterations in larval gut microbiome. While they are key factors in bee health, past studies in social bees show that the gut microbiota function primarily as nutritional mutualists [17] and tend to be distinctly different from those associated with hive-stored pollen [25,39]. Unlike pollen-borne symbionts that are largely eaten and assimilated, the gut microbiota appears to have coevolved with their hosts and thrive in a true mutualism [74]. As further evidence that gut microbiota are not prey, the trophic positions of strict herbivores, even when analysed with their digestive tracts intact (containing microbes) have been reported to be approximately 2.0 [53,75,76]. Moreover, our findings based on trophic biomarkers specifically quantify the contribution of pollen-borne microbes as direct prey for the larvae. Because the gut microbiota function mainly as nutritional mutualists and not as prey, the impact of a disrupted gut microbiome would not be captured within larval trophic biomarker molecules. Based on such collective evidence, it is unlikely that our results could be attributed to the function of the gut microbiome.

Another possible limitation of this work is the chance that sterilizing pollen provisions may have altered pollen quality and compromised the nutritional value of larval diet. However, direct evidence from comparative nutrient analysis revealed that the sterilization procedure did not have any significant effect on the nutritional profile of the pollen provision. Prior research using bumblebees revealed that when recolonized by non-pathogenic microbes, such previously sterilized pollen supported significant gain in colony fitness, implying that the sterilization technique itself did not have a measurable negative outcome for bee health [21]. Given the stability of nitrogen molecules, it is also unlikely that sterilization would have impacted the trophic position of pollen provisions [53,61,77–81].

The nature of bee–microbe symbioses, particularly the extent to which solitary bees rely on pollen-borne symbionts, remains poorly understood. Using multiple lines of evidence, the findings of our study converge to provide strong evidence that pollen-borne microbes are fundamental to bee health. Not only do microbes serve as nutritional mutualists providing bees with adequately fermented and preserved pollen, they also represent a major dietary component for the developing larvae. The loss of these beneficial external microbial symbionts may induce higher mortality among bees by rendering the pollen substrate inadequate for larval consumption, and/or by depriving larvae of essential microbial prey. Given the ubiquity of pollen-associated microbiota, and that nearly all bees rely on these microbial symbionts [48], the findings of this study have profound implications for global bee conservation. Disruptions to this bee–microbe symbiosis by environmental stressors (e.g. elevated concentration of pesticides, heightened burden of parasites and pathogens) may lead to widespread bee losses. To the extent that microbes affect bee survivorship, further research into the role of pollen-associated microbes on larval development will be critical in conserving healthy bee populations.

## Supplementary Material

Supplemental methods and results

## Data accessibility

Detailed methods and additional data are available as the electronic supplementary material.
