# Supplementary material for: Pollen-borne microbes shape bee fitness
Source: Proc Biol Sci. 2019 Jun 12;286(1904):20182894. doi: 10.1098/rspb.2018.2894 (PMC6571465; doi:10.1098/rspb.2018.2894)
Supplement: Supplemental methods and results [file rspb20182894supp1.docx]

**Electronic supplemental material**

**Pollen-borne microbes shape bee fitness**

Prarthana S. Dharampal^1^, Caitlin Carlson^2^, Cameron R. Currie^2^, Shawn A. Steffan^1,3^

^1^Department of Entomology, University of Wisconsin, Madison, Wisconsin, USA

^2^Department of Bacteriology, University of Wisconsin-Madison, Madison, Wisconsin, USA

^3^USDA-ARS, Vegetable Crops Research Unit, Madison, Wisconsin, USA

Corresponding author: [pghosh6@wisc.edu](mailto:pghosh6@wisc.edu)

**Keywords: bee larval survivorship, microbes, pollen provisions, trophic position, trophic biomarker analysis**

S**UPPLEMENTAL MATERIALS AND METHODS**

**1. Bees and pollen provisions**

In April 2017, freshly plugged nesting straws of *Osmia ribifloris* were purchased from a commercial supplier (NativeBees.com). All reeds were collected from a single location in Kaysville, Utah, where the adult females foraged on nectar and pollen from the surrounding trees including Oregon Grape, Asian Pear, and Weeping Cherry. This species was chosen due to several reasons: i) the ease of availability; ii) high number of eggs/nest; iii) high male: female ratio; and iv) comparable morphology and ecology with other congeners including *O. lignaria* [1].

The fresh weight of pollen provisions from each male cell was recorded, and later combined into a single mass to reduce any bias from maternal provisioning. From this collected mass, half of the natural pollen (non-sterile) was stored under aseptic conditions at room temperature, and the rest was sterilized using previously described methods [2]. Sterility of the pollen was verified by the absence of microbial growth after a subsample of the sterilized pollen was plated on general-purpose agar media, and incubated at 28° C for 48 hrs (ESM Figure S2). Comparative proximate analysis (outsourced to University of Wisconsin, Marshfield, Soil and Forage Lab) indicated that sterilization did not have a significant effect on the nutritional composition of pollen provisions (ESM Table S1).

**2. Experimental design**

The experiment consisted of seven treatments, which varied based on the fraction of sterile (SP) versus natural, i.e., non-sterile pollen (NSP). The pollen provision of Treatment 1 was composed entirely of sterile pollen (100%SP); Treatments 2 to 6 consisted of decreasing fractions of sterile pollen; and Treatment 7 consisted of only natural pollen (0%SP). Each treatment was conducted in a single sterile 48 well plate (*N* = 12 larvae/treatment) (ESM Figure S1). Wells were lined using pre-weighed autoclaved tin cups. Pollen provision of each treatment was reconstituted within individual wells under aseptic conditions in a biosafety cabinet. Appropriate amounts of dry sterilized pollen was added to each well, and rehydrated with specific volumes of sterile water to compensate for the moisture loss during sterilization. The volume of water added was based on the pre-determined moisture content of natural pollen provision (ESM Table S2).

**3. Data analysis**

Pearson correlation was used to analyze the relationship between fresh weight of prepupae, and larval developmental time (ESM Figure S3). The effect of diet treatments on these parameters was analyzed using appropriate parametric and non-parametric tests for multiple groups, followed by respective post-hoc comparisons (ESM Figure S4). Difference in larval survivorship across treatments was explored using Kaplan-Meier survival plots and log-rank test (ESM Figure S5). A repeated measures ANOVA was used to test the differences in larval weights across treatments over time. The difference in larval NLFA was explored using multivariate statistical analysis. Larval trophic position (TP) was estimated using compound-specific isotopic analysis of amino acids (CSIA-AA), by measuring the isotopic signatures (‰) of acids, glutamic acid (δ^15^N_glu_) and phenylalanine (δ^15^N_phe_). Previously establish equation for terrestrial, C3 plant-based food-webs was used to calculate larval TP [3,4] ; TP = [(δ^15^N_glu_ – δ^15^N_phe_ + 8.4)/7.2] + 1. TP estimates were analyzed using appropriate parametric tests. All analyses were conducted using SPSS Version 24.0, Armonk, NY.

**SUPPLEMENTAL RESULTS**

The fraction of SP within the pollen provisions had a significant effect on the fresh weight of prepupae (One-way ANOVA, *F*_6,59_ = 32.97, *P* < 0.0001). Tukey post-hoc indicated that larvae raised on 100%SP had significantly lower weight than all other treatments. There was a significant difference in the larval developmental time between the different treatments (Independent samples Kruskal-Wallis test, *H*_6_ = 26.53, *P* < 0.0001). Pearson correlation analysis showed that the %SP within pollen provisions had a significant negative relationship with fresh weight of prepupae (*r* = −0.82, *N* = 66, *P* < 0.0001), and a significant positive relationship with larval developmental time (*r* = 0.48, *N* = 66, *P* < 0.0001). Additionally, across all treatments, larval developmental time was negatively correlated with prepupal fresh weight (*r* = −0.68, *N* = 66, *P* < 0.0001) (ESM Figure S3). Kaplan-Meier survival analysis and log-rank test indicated that there was a significant difference in larval survivorship across treatments (*P* < 0.001) (ESM Figure S5, Table S3). Pairwise comparisons indicated that survivorship was significantly lower among treatments with higher %SP (ESM Figure S4).

For the repeated measures ANOVA of larval weight (across seven treatments and four time points), Greenhouse-Geisser correction (ε = 0.59) was used to adjust for the violation of Mauchly’s test of sphericity, χ^2^ (5) = 47.74, *P* < 0.001). Results showed that there was a significant difference between larval weights across the four time points, (*F*_1.77, 72.62_ = 494.92, *P* < 0.0001), and between treatments (*F*_6, 41_ = 13.14, *P* < 0.0001). There was also a significant interaction between time and treatment (*F*_10.63, 72.62_ = 11.26, *P* < 0.0001). Following up on this interaction indicated that while there was no difference in larval weight at day 1, larvae consuming higher %SP showed lower weight gain as time progressed.

TP of larvae consuming 100%SP was 2.27 ± 0.07, indicating that ~30% of larval diet was of heterotrophic origin. In contrast, larvae consuming 0%SP showed significantly higher TP (2.79 ± 0.07), deriving nearly 80% of proteins from herbivorous heterotrophs. Both groups had significantly higher TPs than expected of strict herbivores (TP_herbivore_ = 2.0). Based on the ubiquity and abundance of microbes within aged pollen substrates, such non-integer trophic identities among bee larvae (2.0 < TP < 3.0) likely results from the assimilation of varying proportions of dietary proteins from both plant (autotrophic), and non-plant (in this case, microbial) resources [5] (ESM Table S4).

The NLFA profiles of larvae raised on 100%SP versus 0%SP showed distinct differences (ESM Table S5, Figure S6). Scatter plot from a principal component analysis revealed that all larvae raised on 100%SP had positive PC1 scores. In particular, this group showed high positive loadings for relative biomarkers associated with plants (16:1ω13t, 18:1ω9) [6,7]. In contrast, larvae raised on 0%SP were significantly more enriched in saturated fatty acids (10:0, 12:0, and 18:0), which can be synthesized *de novo* across several insect taxa (ESM Figure S7) [8]. The ratio of relative fungal and plant biomarkers (18:2ω6/18:1ω9), was significantly lower among larvae raised on 100%SP, indicating greater herbivory among this group, compared to larvae raised on 0%SP, which demonstrated greater fungivory [6]. It should be noted that although bees may be capable of synthesizing select fatty acids, pollen-derived subsidies of 18:1 have been reported to be vital in honey bee nutrition [9]. In contrast, *Osmia* lack the ability to biosynthesizing 18:2, implying that 18:2ω6 may be an appropriate biomarker molecule for their trophic analysis [10]. The sum of bacterial biomarkers (a15:0, i15:0, a17:0, i17:0) was trivial in both groups (< 1% of total NLFAs), although the concentration was nearly double for the larvae raised on 0%SP (0.60 ± 0.46) compared to those raised on 100%SP (0.29 ± 0.08). Moreover, the total abundance of free fatty acids, known to be the primary source of energy for non-feeding pupae [11], was nearly 15 times greater for this group, suggestive of greater fitness among larvae consuming a microbe-rich diet.

1. Sampson BJ, Cane JHB and management potential for three orchard bee specie, Kirker GT, Stringer SJ, Spiers JM. 2009 Biology and management potential for three orchard bee species (Hymenoptera: Megachilidae): Osmia ribifloris Cockerell, O. lignaria (Say) and O.chalybea Smith with emphasis on the former. Acta Hort. 810, 549–555.

2. Steffan SA, Dharampal PS, Diaz-Garcia LA, Currie CR, Zalapa JE, Hittinger CT. 2017 Empirical, metagenomic, and computational techniques illuminate the mechanisms by which fungicides compromise bee health. JoVE 54631, e54631. (doi:10.3791/54631)

3. Steffan SA, Chikaraishi Y, Currie CR, Horn H, Gaines-Day HR, Pauli JN, Zalapa JE, Ohkouchi N. 2015 Microbes are trophic analogs of animals. Proc. Natl. Acad. Sci. 112, 201508782. (doi:10.1073/pnas.1508782112)

4. Chikaraishi Y, Ogawa NO, Doi H, Ohkouchi N. 2011 15N/14N ratios of amino acids as a tool for studying terrestrial food webs: a case study of terrestrial insects (bees, wasps, and hornets). Ecol. Res. 26, 835–844. (doi:10.1007/s11284-011-0844-1)

5. Steffan SA, Chikaraishi Y, Dharampal PS, Pauli JN, Guédot C, Ohkouchi N. 2017 Unpacking brown food-webs: Animal trophic identity reflects rampant microbivory. Ecol. Evol. 7, 3532–3541. (doi:10.1002/ece3.2951)

6. Ruess L, Schütz K, Migge-Kleian S, Häggblom MM, Kandeler E, Scheu S. 2007 Lipid composition of Collembola and their food resources in deciduous forest stands—implications for feeding strategies. Soil Biol. Biochem. 39, 1990–2000. (doi:10.1016/j.soilbio.2007.03.002)

7. Olsson PA. 1999 Signature fatty acids provide tools for determination of the distribution and interactions of mycorrhizal fungi in soil. FEMS Microbiol. Ecol. 29, 303–310. (doi:10.1016/S0168-6496(99)00021-5)

8. Stanley‐Samuelson DW, Jurenka RA, Cripps C, Blomquist GJ, de Renobales M. 1988 Fatty acids in insects: Composition, metabolism, and biological significance. Arch. Insect Biochem. Physiol. 9, 1–33. (doi:10.1002/arch.940090102)

9. Manning R. 2001 Fatty acids in pollen: a review of their importance for honey bees. Bee World 82, 60–75. (doi:10.1080/0005772X.2001.11099504)

10. Cripps C, Blomquist GJ, de Renobales M. 1986 De novo biosynthesis of linoleic acid in insects. Biochim. Biophys. Acta (BBA)/Lipids Lipid Metab. 876, 572–580. (doi:10.1016/0005-2760(86)90046-9)

11. Gilby AR. 1965 Lipids and Their Metabolism in Insects. Annu. Rev. Entomol. 10, 141–160. (doi:10.1146/annurev.en.10.010165.001041)

Figure S1. Schematic representation of experimental design

**SUPPLEMENTAL FIGURES**


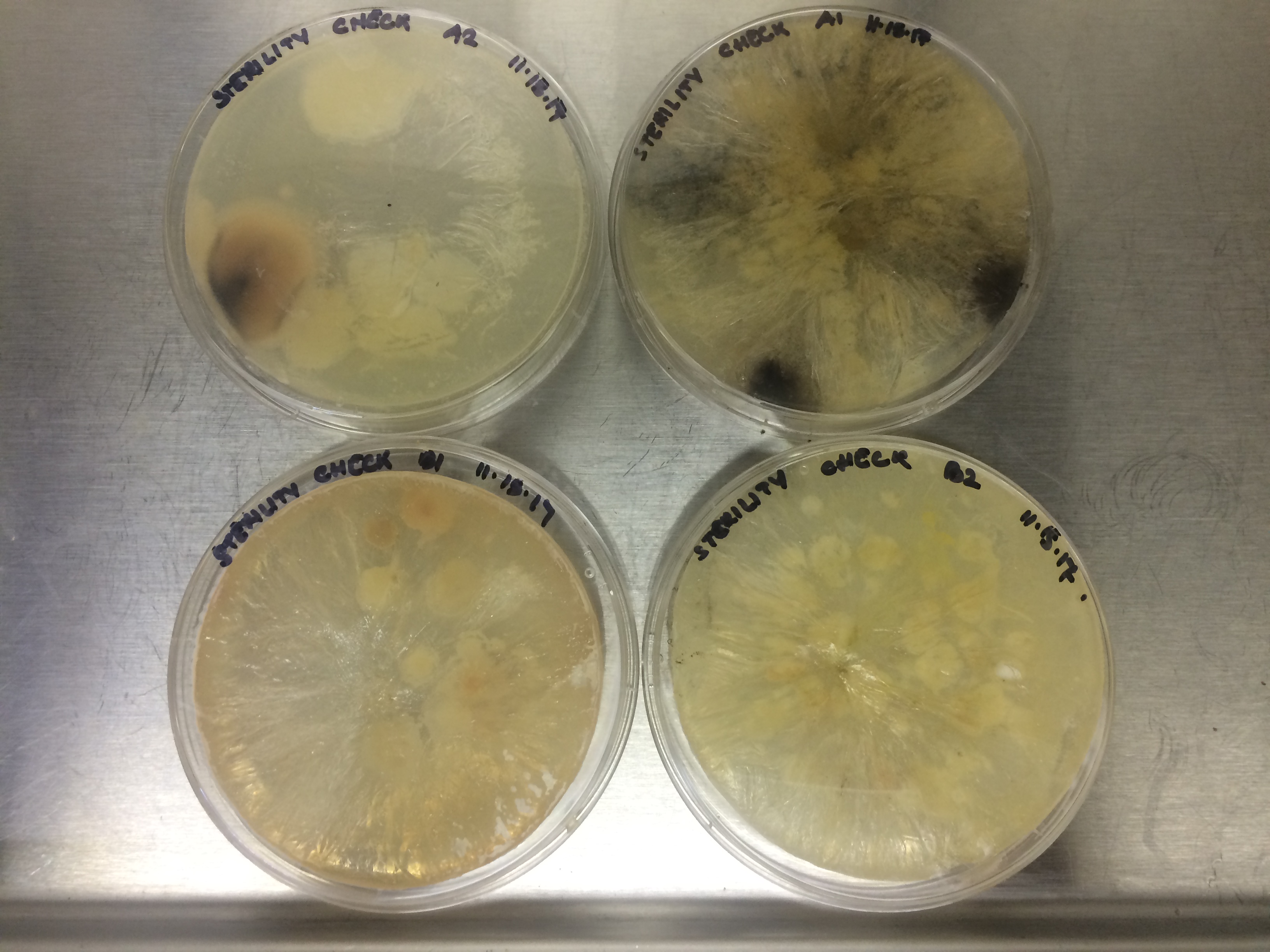

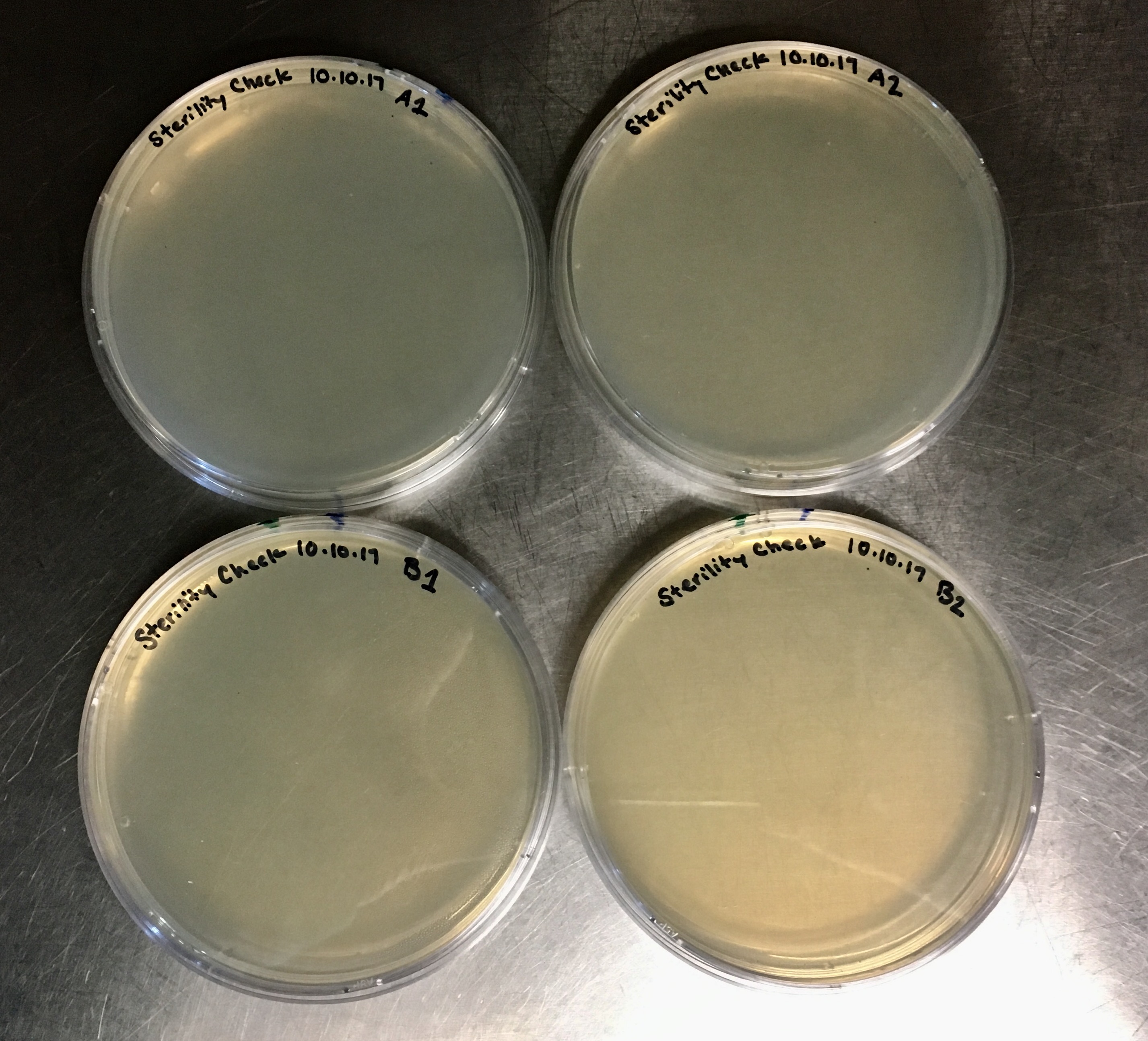


A

B

Figure S2. Yeast malt extract agar plates inoculated with A) 100% sterile pollen; B) 0% sterile pollen, and incubated for 48 hrs at 28° C. The lack of growth in (A) confirmed the absence of microbes within the sterilized pollen

Figure S3. Correlation between (a) percent sterile pollen and fresh weight of prepupae (Pearson’s  *r* = -0.82, *P* < 0.0001); (b) percent sterile pollen and larval developmental time (Pearson’s *r* = 0.48, *P* < 0.0001). *N* = 66.


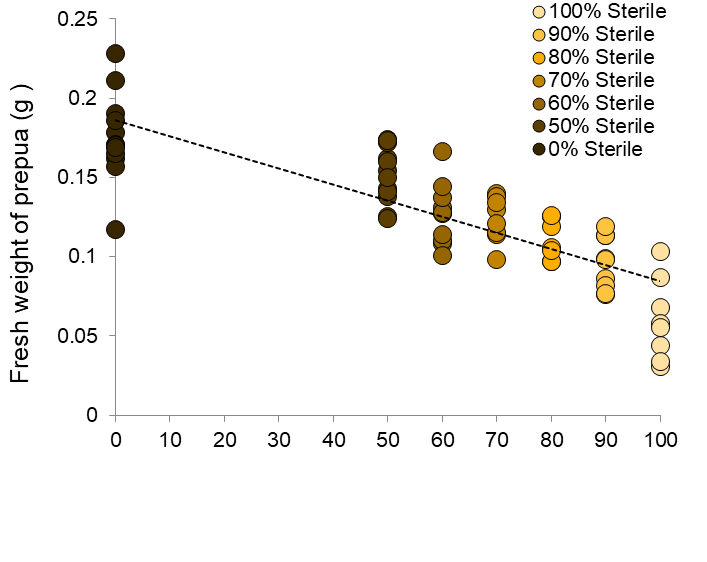

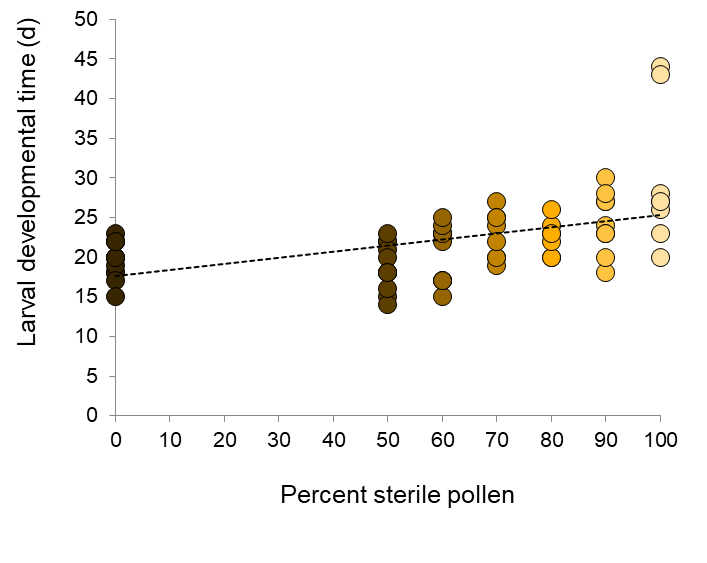


b)

a)

Figure S4. Pair-wise comparisons between a) prepupal fresh weight (one-way ANOVA, Tukey’s post-hoc), b) larval developmental time (Bonferroni corrected Kruskal-Wallis k samples, one-way ANOVA), and c) larval survivorship (Bonferroni corrected log rank test) across treatments. * *P* < 0.05; ** *P* < 0.01; *** *P* < 0.001; **** *P* < 0.0001


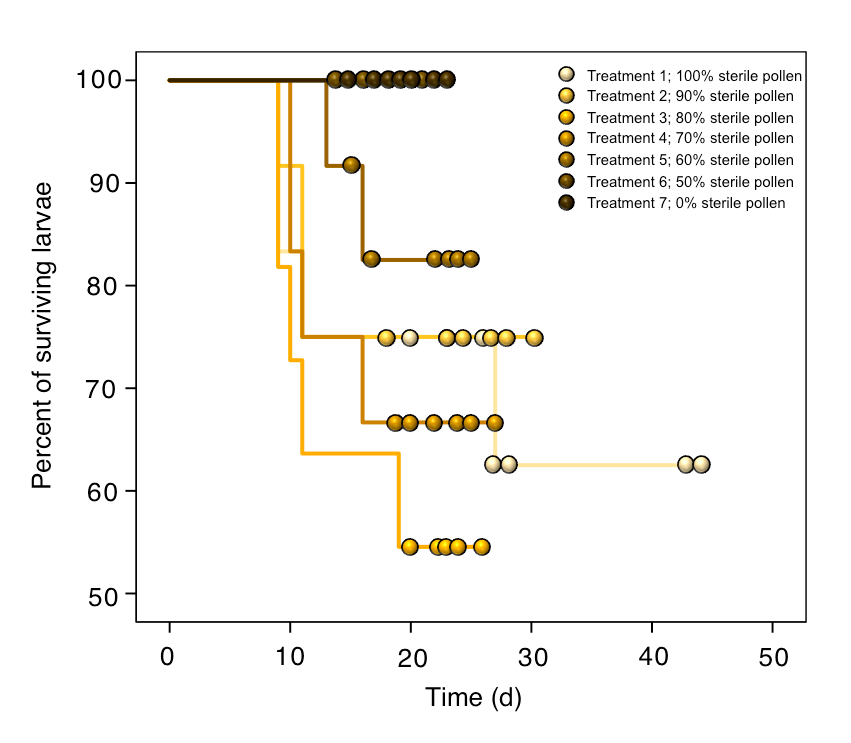


Figure S5. Survival plot of *O. ribifloris* larvae across diet treatments. Circles corresponding to each treatment indicate the time at which individual larva reached the prepupal stage. The log rank test indicated significant differences in survivorship across the seven treatments (*N* = 12 each, *P* < 0.0001).

Figure S6. Relative abundance of NLFAs (percentages of total) from larvae raised on diet treatments (Mean ± 1 SE; *N* = 5 per treatment). Significant differences in abundance are indicated by * (*t-*tests; *P* < 0.05). Marker FAs are in bold.


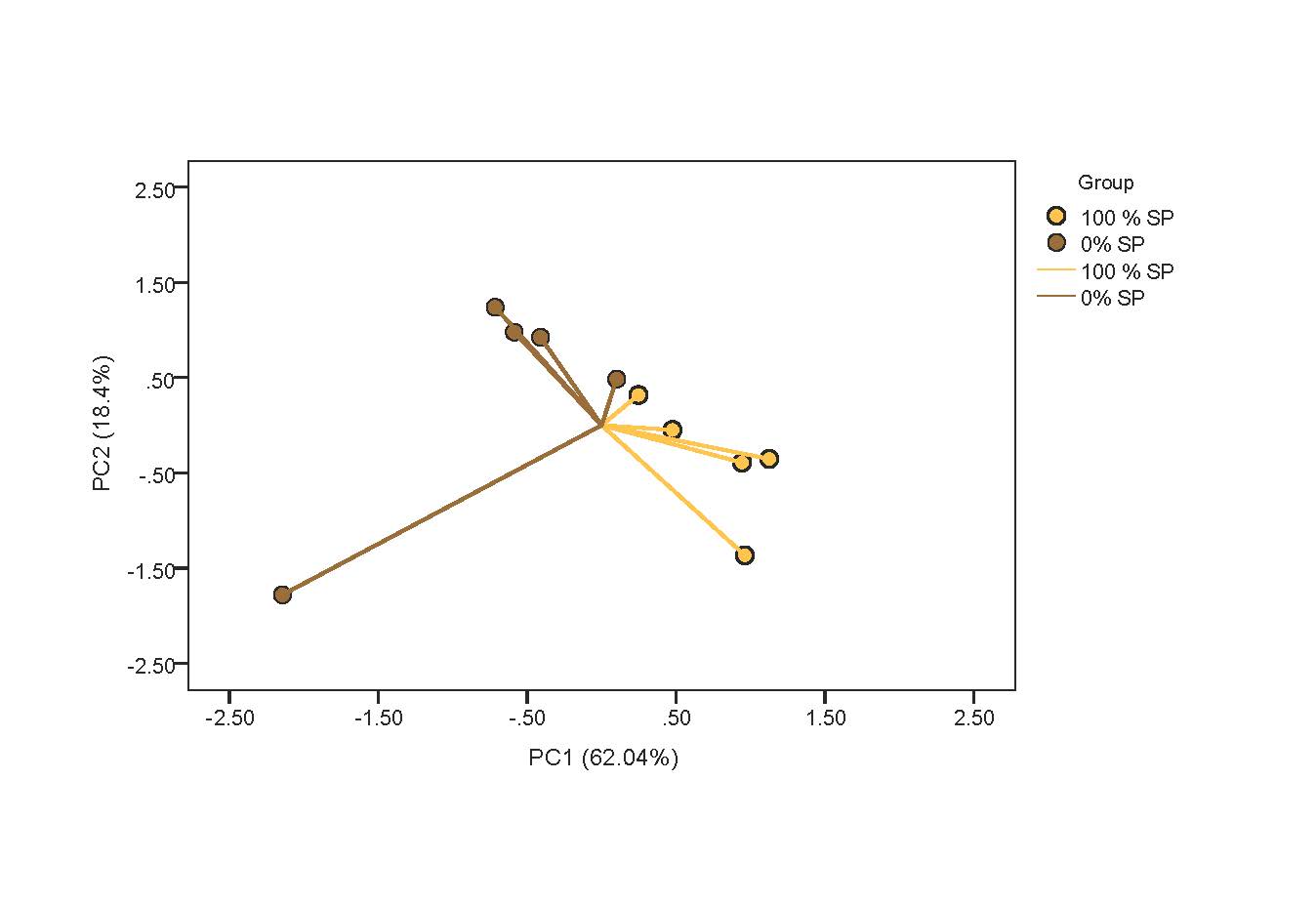


Figure S7. Principle components analysis showing variation in the NLFA compositions of *O. ribifloris* larvae fed on 100%SP and 0%SP. Loadings of positive eigenvalues on PC 1 were of 18:1ω7c, 18:1ω9, 14:1b, 18:1ω5, and 16:1ω13t, and negative eigenvalues were of saturated fatty acids 10:0, 12:0 and 18:0. Loadings of positive eigenvalues on PC 2 were of 14:0, 14:1c and 22:0.

**SUPPLEMENTAL TABLES**

Table S1. Comparative proximate analysis of wild-collected pollen provisions of *Osmia sp.* before (0% Sterile pollen) and after (100% Sterile pollen) sterilization (*N* = 3 each).

|  |  | 0% Sterile pollen | |  | 100% Sterile pollen | |  | *p* value |
| --- | --- | --- | --- | --- | --- | --- | --- | --- |
|  |  | Mean | SE |  | Mean | SE |  |  |
| Dry Matter (DM) | (% as fed) | 88.53 | 36.14 |  | 87.75 | 35.82 |  | 0.47 |
| Moisture | (% as fed) | 11.47 | 4.68 |  | 12.25 | 5.00 |  | 0.47 |
| Crude Protein | (% of DM) | 23.09 | 9.43 |  | 24.63 | 10.05 |  | 0.33 |
| Neutral Detergent Fiber | (% of DM) | 2.12 | 0.86 |  | 2.22 | 0.91 |  | 0.46 |
| Non-Fiber Carbohydrates | (% of DM) | 65.26 | 26.64 |  | 62.91 | 25.68 |  | 0.17 |
| Fat | (% of DM) | 3.87 | 1.58 |  | 4.13 | 1.69 |  | 0.56 |
| Total Digestible Nutrients | (% of DM) | 85.80 | 35.03 |  | 85.54 | 34.92 |  | 0.68 |
| Phosphorus | (% of DM) | 0.10 | 0.04 |  | 0.07 | 0.03 |  | 0.11 |
| Calcium | (% of DM) | 0.09 | 0.04 |  | 0.08 | 0.03 |  | 0.52 |
| Potassium | (% of DM) | 0.61 | 0.25 |  | 0.57 | 0.23 |  | 0.04 |
| Magnesium | (% of DM) | 0.08 | 0.03 |  | 0.08 | 0.03 |  | 0.16 |
| Ash | (% of DM) | 5.66 | 2.31 |  | 6.12 | 2.50 |  | 0.46 |

Table S2. Composition of manipulated pollen provisions across diet treatments

| Treatment | % Non-sterile Pollen | Fresh weight of  natural pollen (g) | % Sterile Pollen | Fresh weight of sterile pollen (g) | Dry weight of sterile pollen (g) | Weight of sterile water added (gm) | Vol. of sterile water added (µl) |
| --- | --- | --- | --- | --- | --- | --- | --- |
| 1 | 0 | 0.000 | 100 | 0.350 | 0.280 | 0.070 | 70 |
| 2 | 10 | 0.035 | 90 | 0.315 | 0.252 | 0.063 | 63 |
| 3 | 20 | 0.070 | 80 | 0.280 | 0.224 | 0.056 | 56 |
| 4 | 30 | 0.105 | 70 | 0.245 | 0.196 | 0.049 | 49 |
| 5 | 40 | 0.140 | 60 | 0.210 | 0.168 | 0.042 | 42 |
| 6 | 50 | 0.175 | 50 | 0.175 | 0.140 | 0.035 | 35 |
| 7 | 100 | 0.350 | 0 | 0.000 | 0.000 | 0.000 | 0 |

Table S3. Description of diet treatments and parameters of larval fitness

|  | **TREATMENT** | | | | | | | |  | |
| --- | --- | --- | --- | --- | --- | --- | --- | --- | --- | --- |
|  | **1** | **2** | **3** | **4** | **5** | **6** | **7** | ***P* value** | |  |
| Percent sterile pollen | 100% | 90% | 80% | 70% | 60% | 50% | 0% |  | |  |
| Wet weight of sterile pollen per provision (g) | 0.350 | 0.315 | 0.280 | 0.245 | 0.210 | 0.175 | 0.000 |  | |  |
| Wet weight of non-sterile pollen per provision (g) | 0.000 | 0.035 | 0.070 | 0.105 | 0.140 | 0.175 | 0.350 |  | |  |
| Percent larval survivorship^1^ | 66.67 | 75.00 | 58.33 | 66.67 | 83.33 | 100.00 | 100.00 | < 0.001 | |  |
| Larval developmental time (d) ^2,3^ | 29.75  (3.14) | 24.44  (1.30) | 22.57  (0.81) | 22.75  (1.03) | 20.60  (1.16) | 18.75  (0.84) | 19.67  (0.69) | < 0.0001 | |  |
| Fresh weight of prepupa (g) ^2,4^ | 0.06  (0.01) | 0.10  (0.01) | 0.11  (0.00) | 0.12  (0.01) | 0.13  (0.01) | 0.15  (0.01) | 0.18  (0.01) | < 0.0001 | |  |

^1^Kaplan-Meier analysis, log-rank test ^2^Mean (± 1SE)

^3^Kruskal-Wallis test ^4^One-way ANOVA

Table S4. δ^15^N signature of glutamic acid (glu) and phenylalanine (phe) of *Osmia* larvae raised on sterile (100% Sterile pollen) and natural (0% Sterile pollen) pollen provisions (*N* = 3 per treatment).

|  | | | 100% Sterile pollen | | | | | |  | | 0% Sterile pollen | | | | | |
| --- | --- | --- | --- | --- | --- | --- | --- | --- | --- | --- | --- | --- | --- | --- | --- | --- |
|  |  |  | Rep 1 | | Rep 2 | | Rep 3 | |  | | Rep 1 | | Rep 2 | | Rep 3 | |
| δ^15^N  (‰, vs Air) | Glutamic acid | 1.91 | | 2.22 | | 3.48 | |  | | 4.87 | | 5.72 | | 5.87 | |  |
|  | Phenylalanine | 2.12 | | 1.34 | | 1.98 | |  | | 1.22 | | 1.22 | | 0.53 | |  |
| Mean TP (± 1 SE) ^1, 2^ | | | 2.27 (0.07)^3^ | | | | | |  | | 2.79 (0.07)^4^ | | | | | |

^1^ Trophic position (TP) = [{(δ^15^N_glu_ - δ^15^N_phe_ + 8.4)/ 7.2} +1]

^2^ *P =* 0.006 (Independent samples *t*-test, *t*_4_ = -5.40)

^3^ *P =* 0.03 (One sample, one-tailed *t*-test, Test value = 2.0, *t*_2_ = 3.86)

^4^ *P =* 0.003 (One sample, one-tailed *t*-test, Test value = 2.0, *t*_2_ = 11.69)

Table S5. Fatty acid methyl ester and free fatty acid profile (mg gdw^-1^) of *Osmia* prepupae (*N* = 5 per treatment)

|  |  | 100% Sterile pollen | |  | 0% Sterile pollen | |
| --- | --- | --- | --- | --- | --- | --- |
|  |  | Mean | ± SE |  | Mean | ± SE |
| Saturated FAMEs | 10:0 | 0.28 | 0.17 |  | 0.23 | 0.09 |
|  | 12:0 | 2.08 | 1.15 |  | 2.07 | 0.55 |
|  | 14:0 | 5.52 | 1.31 |  | 8.11 | 2.87 |
|  | i15:0^1^ | 0.01 | 0.01 |  | 0.08 | 0.08 |
|  | a15:0^1^ | ND |  |  | 0.04 | 0.04 |
|  | 15:0 | 0.03 | 0.02 |  | 0.01 | 0.01 |
|  | 16:0 | 13.06 | 2.48 |  | 12.10 | 6.21 |
|  | i17:0^1^ | 0.06 | 0.03 |  | 0.03 | 0.03 |
|  | a17:0^1^ | 0.32 | 0.09 |  | 0.14 | 0.05 |
|  | 17:0 | 0.16 | 0.05 |  | 0.05 | 0.02 |
|  | 18:0 | 1.85 | 0.32 |  | 3.30 | 0.43 |
|  | 19:0 | 0.03 | 0.01 |  | 0.02 | 0.01 |
|  | 20:0 | 0.19 | 0.07 |  | 0.14 | 0.04 |
|  | 21:0 | 0.03 | 0.01 |  | 0.00 | 0.00 |
|  | 22:0 | 0.07 | 0.04 |  | 0.05 | 0.03 |
|  | 24:0 | 0.06 | 0.03 |  | 0.01 | 0.01 |
| Monoenoic FAMEs | 14:1a | 0.07 | 0.01 |  | 0.02 | 0.02 |
|  | 14:1b | 0.21 | 0.02 |  | 0.14 | 0.08 |
|  | 14:1c | 11.41 | 2.38 |  | 15.92 | 5.79 |
|  | 16:1ω5 | 1.13 | 0.19 |  | 0.86 | 0.37 |
|  | 16:1ω7c | 50.80 | 9.15 |  | 40.01 | 16.58 |
|  | 16:1ω7t | 0.80 | 0.23 |  | 0.47 | 0.23 |
|  | 16:1ω9 | 0.82 | 0.07 |  | 0.21 | 0.13 |
|  | 16:1ω13t | 0.25 | 0.04 |  | 0.09 | 0.05 |
|  | 18:1ω5 | 0.36 | 0.07 |  | 0.16 | 0.07 |
|  | 18:1ω7c | 7.91 | 1.01 |  | 3.27 | 1.47 |
|  | 18:1ω7t | 0.37 | 0.07 |  | 0.13 | 0.06 |
|  | 18:1ω9^3^ | 21.72 | 3.37 |  | 10.70 | 4.81 |
|  | 20:1ω7 | 0.09 | 0.01 |  | 0.01 | 0.01 |
|  | 20:1ω9 | 0.13 | 0.02 |  | 0.03 | 0.02 |
|  | 20:1ω11 | 0.15 | 0.03 |  | 0.06 | 0.02 |
| Polyenoic FAMEs | 16:2ω4 | 0.08 | 0.01 |  | 0.04 | 0.03 |
|  | 18:2ω6^2^ | 4.20 | 0.58 |  | 3.09 | 1.20 |
|  | 18:3ω3 | 9.37 | 1.29 |  | 7.45 | 2.82 |
|  | 18:3ω6 | 0.12 | 0.04 |  | ND |  |
|  | 18:4ω3 | 0.09 | 0.03 |  | ND |  |
|  | 20:2? | 0.03 | 0.01 |  | 0.01 | 0.01 |
| Free fatty acids | 12:0 | ND |  |  | 0.04 | 0.04 |
|  | 14:0 | 0.05 | 0.03 |  | 4.04 | 1.14 |
|  | 14:1 | 0.08 | 0.04 |  | 6.09 | 1.95 |
|  | 16:0 | 1.04 | 0.42 |  | 10.26 | 2.03 |
|  | 16:1 | 2.14 | 0.83 |  | 22.54 | 5.57 |
|  | 18:1 | ND |  |  | 5.09 | 1.54 |
| ∑FAMEs | | 133.83 | 23.37 |  | 109.02 | 42.97 |
| ∑Absolute bacterial biomarkers | | 0.38 | 0.11 |  | 0.29 | 0.16 |
| ∑Free fatty acids^4^ | | 16.55 | 1.31 |  | 240.31 | 12.13 |
| Fungal: Plant FAMEs^5, 6^ | | 0.20 | 0.02 |  | 0.31 | 0.01 |
|  | |  |  |  |  |  |

^1^ Absolute bacterial biomarkers ^2^ Relative fungal biomarker

^3^ Relative plant biomarker ^4^ *P* < 0.01 (Mann-Whitney *U* = 25.0)

^5^ *P* < 0.01(*t*_8_ = -3.67) ^6^ Calculated as ratio of 18:2ω6: 18:1ω9

ND: Not detected
